# Supplementary material for: The CDK Pef1 and protein phosphatase 4 oppose each other for regulating cohesin binding to fission yeast chromosomes
Source: eLife. 2020 Jan 2;9:e50556. doi: 10.7554/eLife.50556 (PMC6954021; doi:10.7554/eLife.50556)
Supplement: Supplementary file 1. [file elife-50556-supp1.docx]

| Key Resources Table | | | | |
| --- | --- | --- | --- | --- |
| Reagent type (species) or resource | Designation | Source or reference | Identifiers | Additional information |
| Strain, strain background (*S. pombe*) | *h^-^* | This study | 2 | J.P.J. Lab. Strain collection |
| Strain, strain background (*S. pombe*) | *h^+^*/*h^-^* *leu1/leu1 ura4-D18/ura4-D18 ade6-210/ade6-216 ssl3*Δ::*ura4*^+^*/ssl3*^+^ | This study | 962 | J.P.J. Lab. Strain collection |
| Strain, strain background (*S. pombe*) | *h^-^* *rad21-9PK-kan*^R^ *psm3*^K105NK106N^ | This study | 1004 | J.P.J. Lab. Strain collection |
| Strain, strain background (*S. pombe*) | *h^-^* *mis4-367* | This study | 2729 | J.P.J. Lab. Strain collection |
| Strain, strain background (*S. pombe*) | *h^+^* *mis4-367* | This study | 2730 | J.P.J. Lab. Strain collection |
| Strain, strain background (*S. pombe*) | *h^-^* *leu1* *mis4-3HA-LEU2* | This study | 2804 | J.P.J. Lab. Strain collection |
| Strain, strain background (*S. pombe*) | *h^-^* *wpl1*Δ::*kan*^R^ | This study | 3060 | J.P.J. Lab. Strain collection |
| Strain, strain background (*S. pombe*) | *h^-^* *mis4-367* *wpl1*Δ::*kan*^R^ | This study | 3474 | J.P.J. Lab. Strain collection |
| Strain, strain background (*S. pombe*) | *h^-^* *ura4-D18* *pas1*Δ::*ura4*^+^ | PMID: 10982385 | 3637 | J.P.J. Lab. Strain collection |
| Strain, strain background (*S. pombe*) | *h^-^* *ura4-Δ18* *mis4-367* *pef1*Δ::*ura4*^+^ | This study | 3651 | J.P.J. Lab. Strain collection |
| Strain, strain background (*S. pombe*) | *h^+^* *ura4-D18* *mis4-367* *pef1*Δ::*ura4*^+^ | This study | 3652 | J.P.J. Lab. Strain collection |
| Strain, strain background (*S. pombe*) | *h^-^* *rad21-9PK-kan*^R^ | This study | 3789 | J.P.J. Lab. Strain collection |
| Strain, strain background (*S. pombe*) | *h^-^* *mis4-367 rad21-9PK-kan*^R^ | This study | 3791 | J.P.J. Lab. Strain collection |
| Strain, strain background (*S. pombe*) | *h^+^* | This study | 3820 | J.P.J. Lab. Strain collection |
| Strain, strain background (*S. pombe*) | *h^+^*/*h^-^* *leu1/leu1*^+^ *ura4-D18/ura4-D18 ade6-210/ade6-216* *mis4*^+^/*mis4*Δ::*kan*^R^ *pef1*Δ::*ura4*^+^/ *pef1*Δ::*ura4*^+^ | This study | 3982 | J.P.J. Lab. Strain collection |
| Strain, strain background (*S. pombe*) | *h^-^* *cdc10-129 rad21-9PK-kan*^R^ | This study | 3984 | J.P.J. Lab. Strain collection |
| Strain, strain background (*S. pombe*) | *h^-^* *cdc10-129* | This study | 3985 | J.P.J. Lab. Strain collection |
| Strain, strain background (*S. pombe*) | *h- cdc25-22 pef1-as rad21-9PK-kanR* | This study | 4822 | J.P.J. Lab. Strain collection |
| Strain, strain background (*S. pombe*) | *h- ura4-D18 mis4-367 swi6Δ::ura4+* | This study | 5124 | J.P.J. Lab. Strain collection |
| Strain, strain background (*S. pombe*) | *h- his1-102 swi6Δ::his1+* | This study | 5584 | J.P.J. Lab. Strain collection |
| Strain, strain background (*S. pombe*) | *h^+^* *eso1-H17* | This study | 5912 | J.P.J. Lab. Strain collection |
| Strain, strain background (*S. pombe*) | *h^-^* *eso1-H17* | This study | 5913 | J.P.J. Lab. Strain collection |
| Strain, strain background (*S. pombe*) | *h^+^* *pph3*Δ::*hyg*^R^ | This study | 6095 | J.P.J. Lab. Strain collection |
| Strain, strain background (*S. pombe*) | *h^-^* *mis4-367 pph3*Δ::*hyg*^R^ | This study | 6209 | J.P.J. Lab. Strain collection |
| Strain, strain background (*S. pombe*) | *h^-^* *mis4-367 pef1*Δ::*ura4*^+^ *pph3*Δ::*hyg*^R^ *ura4-D18* | This study | 6212 | J.P.J. Lab. Strain collection |
| Strain, strain background (*S. pombe*) | *h^-^* *cdc10-129 rad21-9PK-kan*^R^ *pef1*Δ::*ura4*^+^ *ura4-D18* | This study | 6228 | J.P.J. Lab. Strain collection |
| Strain, strain background (*S. pombe*) | *h^+^* *ura4-D18* *pef1*Δ::*ura4*^+^ | This study | 6246 | J.P.J. Lab. Strain collection |
| Strain, strain background (*S. pombe*) | *h^-^* *cdc10-129* *ura4-D18* *pef1*Δ::*ura4*^+^ *rad21FLAG3-kan*^R^ | This study | 6360 | J.P.J. Lab. Strain collection |
| Strain, strain background (*S. pombe*) | *h^-^* *cdc10-129* *rad21FLAG3-kan*^R^ | This study | 6393 | J.P.J. Lab. Strain collection |
| Strain, strain background (*S. pombe*) | *h^-^* *pef1-GFP-nat*^R^ | This study | 6400 | J.P.J. Lab. Strain collection |
| Strain, strain background (*S. pombe*) | *h^-^* *pef1*Δ::*nat*^R^ | This study | 6638 | J.P.J. Lab. Strain collection |
| Strain, strain background (*S. pombe*) | *h^+^* *pef1*Δ::*hyg*^R^ | This study | 6645 | J.P.J. Lab. Strain collection |
| Strain, strain background (*S. pombe*) | *h^-^* *pef1*Δ::*hyg*^R^ | This study | 6646 | J.P.J. Lab. Strain collection |
| Strain, strain background (*S. pombe*) | *h^-^* *pef1-F78A* | This study | 6699 | J.P.J. Lab. Strain collection |
| Strain, strain background (*S. pombe*) | *h^-^rad21-9PK-kan*^R^ *pef1-F78A* | This study | 6701 | J.P.J. Lab. Strain collection |
| Strain, strain background (*S. pombe*) | *h^-^* *eso1-H17* *pef1*Δ::*hyg*^R^ | This study | 6724 | J.P.J. Lab. Strain collection |
| Strain, strain background (*S. pombe*) | *h^+^* *eso1-H17* *pef1*Δ::*hyg*^R^ | This study | 6725 | J.P.J. Lab. Strain collection |
| Strain, strain background (*S. pombe*) | *h^-^* *mis4-367* *pef1-F78A* | This study | 6747 | J.P.J. Lab. Strain collection |
| Strain, strain background (*S. pombe*) | *h^-^* *mis4-367* *pef1*Δ::*hyg*^R^ | This study | 6753 | J.P.J. Lab. Strain collection |
| Strain, strain background (*S. pombe*) | *h^-^* *ura4*^+^-*tet*^ON^ | This study | 6777 | J.P.J. Lab. Strain collection |
| Strain, strain background (*S. pombe*) | *h^-^* *mis4-367 pef1*Δ::*nat*^R^ | This study | 6849 | J.P.J. Lab. Strain collection |
| Strain, strain background (*S. pombe*) | *h^-^* *mis4-367 rad21-9PK-kan*^R^ *pef1-F78A* | This study | 6923 | J.P.J. Lab. Strain collection |
| Strain, strain background (*S. pombe*) | *psm3-gly11-TEV3-rad21-kanR* *pef1*Δ::*nat*^R^ | This study | 6926 | J.P.J. Lab. Strain collection |
| Strain, strain background (*S. pombe*) | *psm3-gly11-TEV3-rad21-kanR* | This study | 6928 | J.P.J. Lab. Strain collection |
| Strain, strain background (*S. pombe*) | *h^-^* *mis4-367 psm3-gly11-TEV3-rad21-kanR* *pef1*Δ::*nat*^R^ | This study | 6929 | J.P.J. Lab. Strain collection |
| Strain, strain background (*S. pombe*) | *h^-^* *leu1* *pef1-GFP-nat*^R^ *mis4-3HA-LEU2* | This study | 6940 | J.P.J. Lab. Strain collection |
| Strain, strain background (*S. pombe*) | *h^-^* *mis4-367 psm3-gly11-TEV3-rad21-kanR* | This study | 6943 | J.P.J. Lab. Strain collection |
| Strain, strain background (*S. pombe*) | *h^-^ ura4-D18 eso1*Δ::*ura4*^+^ *rad21*Δ::*nat*^R^ *psm3-gly11-TEV3-rad21-kanR* | This study | 6959 | J.P.J. Lab. Strain collection |
| Strain, strain background (*S. pombe*) | *h^-^ ura4-D18 eso1*Δ::*ura4*^+^ *wpl1*Δ::*hyg*^R^ *rad21*Δ::*nat*^R^ *psm3-gly11-TEV3-rad21-kanR* | This study | 6961 | J.P.J. Lab. Strain collection |
| Strain, strain background (*S. pombe*) | *h^-^ ura4-Δ18 eso1*Δ::*ura4*^+^ *wpl1*Δ::*hyg*^R^ | This study | 6979 | J.P.J. Lab. Strain collection |
| Strain, strain background (*S. pombe*) | *h^-^* *rad21-9PK-kanR* *pef1*Δ::*nat*^R^ | This study | 7411 | J.P.J. Lab. Strain collection |
| Strain, strain background (*S. pombe*) | *h^-^rad21-9PK-kan*^R^ *ura4-D18 eso1*Δ::*ura4*^+^ *wpl1*Δ::*hyg*^R^ | This study | 7512 | J.P.J. Lab. Strain collection |
| Strain, strain background (*S. pombe*) | *h^-^* *ura4*^+^-*tet*^ON^ *SPNCRNA.458*::*hyg*^R^-*tet07*-*rad21FLAG3-kanR* | This study | 7548 | J.P.J. Lab. Strain collection |
| Strain, strain background (*S. pombe*) | *h^-^* *ura4*^+^-*tet*^ON^ *SPNCRNA.458*::*hyg*^R^-*tet07*-*rad21FLAG3-kanR rad21*Δ::*ura4*^+^ | This study | 7567 | J.P.J. Lab. Strain collection |
| Strain, strain background (*S. pombe*) | *h^-^* *ura4*^+^-*tet*^ON^ *SPNCRNA.458*::*hyg*^R^-*tet07*-*rad21FLAG3-kanR rad21*Δ::*ura4*^+^ *mis4-367* | This study | 7580 | J.P.J. Lab. Strain collection |
| Strain, strain background (*S. pombe*) | *h^-^* *ura4*^+^-*tet*^ON^ *SPNCRNA.458*::*hyg*^R^-*tet07*-*rad21FLAG3-kanR rad21*Δ::*ura4*^+^ *mis4-367 pef1*Δ::*nat*^R^ | This study | 7598 | J.P.J. Lab. Strain collection |
| Strain, strain background (*S. pombe*) | *h^+^* *mis4-242* *pef1*Δ::*nat*^R^ | This study | 7638 | J.P.J. Lab. Strain collection |
| Strain, strain background (*S. pombe*) | *h^+^* *mis4-242* | This study | 7639 | J.P.J. Lab. Strain collection |
| Strain, strain background (*S. pombe*) | *h^-^* *cdc10-129* *pef1-GFP-nat*^R^ *ura4-D18* | This study | 7753 | J.P.J. Lab. Strain collection |
| Strain, strain background (*S. pombe*) | *h^-^* *mis4-367 pef1-F78A cdc10-129 NCRNA458::hygR-tet07-rad21-FLAG3-kanR ura4+-TetON* | This study | 7835 | J.P.J. Lab. Strain collection |
| Strain, strain background (*S. pombe*) | *h- pef1-as cdc10-129 rad21-9PK-kanR* | This study | 7964 | J.P.J. Lab. Strain collection |
| Strain, strain background (*S. pombe*) | *h^+^* *leu1-32* *ssl3*Δ::*ura4*^+^ *ars1-ssl3-29*^ts^-*LEU2* | This study | 8112 | J.P.J. Lab. Strain collection |
| Strain, strain background (*S. pombe*) | *h^+^* *leu1-32* *ssl3*Δ::*ura4*^+^ *ars1-ssl3-29*^ts^-*LEU2* *pef1*Δ::*hyg*^R^ | This study | 8114 | J.P.J. Lab. Strain collection |
| Strain, strain background (*S. pombe*) | *h^+^* *pph3*Δ::*hyg*^R^ *pef1*Δ::*kan*^R^ | This study | 9453 | J.P.J. Lab. Strain collection |
| Strain, strain background (*S. pombe*) | *h^-^ pph3*Δ::*hyg*^R^ *psm3^K105NK106N^* | This study | 9520 | J.P.J. Lab. Strain collection |
| Strain, strain background (*S. pombe*) | h- *pph3*Δ::*hyg*^R^ *psm3^K105NK106N^* *mis4^I803M^* | This study | 9590 | J.P.J. Lab. Strain collection |
| Strain, strain background (*S. pombe*) | *h^+^* *pph3*Δ::*hyg*^R^ *psm3^K105NK106N^ mis4^I803M^* | This study | 9591 | J.P.J. Lab. Strain collection |
| Strain, strain background (*S. pombe*) | *h^+^* *pph3*Δ::*hyg*^R^ *psm3^K105NK106N^* | This study | 9664 | J.P.J. Lab. Strain collection |
| Strain, strain background (*S. pombe*) | *h^-^* *cdc10-129* *leu1-32 mis4-GFP-LEU2* | This study | 9748 | J.P.J. Lab. Strain collection |
| Strain, strain background (*S. pombe*) | *h^-^* *psm3*^K105NK106N^ *pph3*Δ::*hyg*^R^ *pas1*Δ::*nat*^R^ | This study | 9806 | J.P.J. Lab. Strain collection |
| Strain, strain background (*S. pombe*) | *h^-^* *psm3*^K105NK106N^ *pph3*Δ::*hyg*^R^ *pef1*Δ::*nat*^R^ | This study | 9849 | J.P.J. Lab. Strain collection |
| Strain, strain background (*S. pombe*) | *h- ura4-D18 mis4-367 pef1Δ::natR swi6Δ::ura4* | This study | 9853 | J.P.J. Lab. Strain collection |
| Strain, strain background (*S. pombe*) | *h- ura4-D18 pef1Δ::natR swi6Δ::ura4+* | This study | 9855 | J.P.J. Lab. Strain collection |
| Strain, strain background (*S. pombe*) | *h^+^*/*h^-^* *leu1/leu1 ura4-D18/ura4-D18 ade6-210/ade6-216 ssl3*Δ::*ura4*^+^*/ssl3*^+^ *pef1*Δ::*nat*^R^/*pef1*^+^ | This study | 9922 | J.P.J. Lab. Strain collection |
| Strain, strain background (*S. pombe*) | *h^+^*/*h^-^* *leu1/leu1 ura4-D18/ura4-D18 ade6-210/ade6-216 ssl3*Δ::*ura4*^+^*/ssl3*^+^ *wpl1*Δ::*kan*^R^/*wpl1*^+^ | This study | 9923 | J.P.J. Lab. Strain collection |
| Strain, strain background (*S. pombe*) | *h^-^* *clg1*Δ::*kan*^R^ | This study | 9959 | J.P.J. Lab. Strain collection |
| Strain, strain background (*S. pombe*) | *h^-^* *psl1*Δ::*kan*^R^ | This study | 9967 | J.P.J. Lab. Strain collection |
| Strain, strain background (*S. pombe*) | *h^-^* *mis4-367* *clg1*Δ::*kan*^R^ | This study | 9993 | J.P.J. Lab. Strain collection |
| Strain, strain background (*S. pombe*) | *h^-^* *mis4-367 psl1*Δ::*kan*^R^ | This study | 9995 | J.P.J. Lab. Strain collection |
| Strain, strain background (*S. pombe*) | *h^-^* *psm3*^K105NK106N^ *pph3*Δ::*hyg*^R^ *clg1*Δ::*kan*^R^ | This study | 10030 | J.P.J. Lab. Strain collection |
| Strain, strain background (*S. pombe*) | *h^-^* *psm3*^K105NK106N^ *pph3*Δ::*hyg*^R^ *psl1*Δ::*kan*^R^ | This study | 10032 | J.P.J. Lab. Strain collection |
| Strain, strain background (*S. pombe*) | *h^-^* *psm3*^K105NK106N^ *pph3*Δ::*hyg*^R^ | This study | 10034 | J.P.J. Lab. Strain collection |
| Strain, strain background (*S. pombe*) | *h^-^* *rad21-9PK-kan*^R^ *psm3*^K105NK106N^ *pph3*Δ::*hyg*^R^ *pef1*Δ::*nat*^R^ | This study | 10035 | J.P.J. Lab. Strain collection |
| Strain, strain background (*S. pombe*) | *h^-^* *psl1-pef1-GFP-kan*^R^ *pef1*Δ::*ura4*^+^ *ura4-D18* | This study | 10083 | J.P.J. Lab. Strain collection |
| Strain, strain background (*S. pombe*) | *h^-^* *cdc10-129* *pef1-GFP-nat*^R^ *pas1*Δ::*kan*^R^ | This study | 10129 | J.P.J. Lab. Strain collection |
| Strain, strain background (*S. pombe*) | h- *h^-^* *pef1-GFP-nat*^R^ *ura4-D18* *pas1*Δ::*ura4*^+^ | This study | 10135 | J.P.J. Lab. Strain collection |
| Strain, strain background (*S. pombe*) | *h^+^* *psm3*^K105NK106N^ *pph3*Δ::*hyg*^R^ | This study | 10150 | J.P.J. Lab. Strain collection |
| Strain, strain background (*S. pombe*) | *h^-^* *psm3*^K105NK106N^ *rad21-T262A* | This study | 10151 | J.P.J. Lab. Strain collection |
| Strain, strain background (*S. pombe*) | *h^+^* *psm3*^K105NK106N^ *pph3*Δ::*hyg*^R^ *rad21-T262A* | This study | 10153 | J.P.J. Lab. Strain collection |
| Strain, strain background (*S. pombe*) | *h^+^* *psm3*^K105NK106N^ *rad21-T262D* | This study | 10154 | J.P.J. Lab. Strain collection |
| Strain, strain background (*S. pombe*) | *h^+^* *psm3*^K105NK106N^ *pph3*Δ::*hyg*^R^ *rad21-T262D* | This study | 10156 | J.P.J. Lab. Strain collection |
| Strain, strain background (*S. pombe*) | *h^+^* *psm3*^K105NK106N^ *rad21-T262E* | This study | 10158 | J.P.J. Lab. Strain collection |
| Strain, strain background (*S. pombe*) | *h^+^* *psm3*^K105NK106N^ *pph3*Δ::*hyg*^R^ *rad21-T262E* | This study | 10160 | J.P.J. Lab. Strain collection |
| Strain, strain background (*S. pombe*) | *h^-^* *cdc10-129* *pef1-GFP-nat*^R^ *psl1*Δ::*kan*^R^ | This study | 10161 | J.P.J. Lab. Strain collection |
| Strain, strain background (*S. pombe*) | h- *h^-^* *pef1-GFP-nat*^R^ *psl1*Δ::*kan*^R^ | This study | 10163 | J.P.J. Lab. Strain collection |
| Strain, strain background (*S. pombe*) | *h^-^* *cdc10-129* *pef1-GFP-nat*^R^ *clg1*Δ::*kan*^R^ | This study | 10180 | J.P.J. Lab. Strain collection |
| Strain, strain background (*S. pombe*) | h- *h^-^* *pef1-GFP-nat*^R^ *clg1*Δ::*kan*^R^ | This study | 10184 | J.P.J. Lab. Strain collection |
| Strain, strain background (*S. pombe*) | *h^-^* *mis4-367 rad21-T262D* | This study | 10191 | J.P.J. Lab. Strain collection |
| Strain, strain background (*S. pombe*) | *h^-^* *mis4-367 rad21-T262E* | This study | 10192 | J.P.J. Lab. Strain collection |
| Strain, strain background (*S. pombe*) | *h^-^* *mis4-367 rad21-T262A* | This study | 10202 | J.P.J. Lab. Strain collection |
| Strain, strain background (*S. pombe*) | *h^-^* *cdc10-129 leu1* *mis4-GFP-LEU2 rad21-T262A* | This study | 10258 | J.P.J. Lab. Strain collection |
| Strain, strain background (*S. pombe*) | *h^-^* *rad21-9PK-kan*^R^ *psm3*^K105NK106N^ *pef1*Δ::*nat*^R^ | This study | 10277 | J.P.J. Lab. Strain collection |
| Strain, strain background (*S. pombe*) | *h^-^* *cdc10-129* *leu1-32 mis4-GFP-LEU2* *pef1*Δ::*hyg*^R^ | This study | 10289 | J.P.J. Lab. Strain collection |
| Strain, strain background (*S. pombe*) | *h^-^* *mis4-367 rad21-T262A* *psl1*Δ::*kan*^R^ | This study | 10304 | J.P.J. Lab. Strain collection |
| Strain, strain background (*S. pombe*) | *h^-^* *mis4-367* *pas1*Δ::*nat*^R^ | This study | 10385 | J.P.J. Lab. Strain collection |
| Strain, strain background (*S. pombe*) | *h^-^* *mis4-367* *rad21-T262D* *psl1*Δ::*kan*^R^ | This study | 10428 | J.P.J. Lab. Strain collection |
| Strain, strain background (*S. pombe*) | *h^-^* *mis4-367 pph3*Δ::*hyg*^R^ *rad21-T262A* | This study | 10430 | J.P.J. Lab. Strain collection |
| Strain, strain background (*S. pombe*) | *h^-^* *mis4-367 pef1*Δ::*nat*^R^ *pph3*Δ::*hyg*^R^ *rad21-T262A* | This study | 10432 | J.P.J. Lab. Strain collection |
| Strain, strain background (*S. pombe*) | *h^-^* *cdc10-129 leu1* *mis4-GFP-LEU2 pef1*Δ::*hyg*^R^ *rad21-T262A* | This study | 10446 | J.P.J. Lab. Strain collection |
| Strain, strain background (*S. pombe*) | *h^-^* *mis4-367 rad21-T262E* *psl1*Δ::*kan*^R^ | This study | 10464 | J.P.J. Lab. Strain collection |
| Strain, strain background (*S. pombe*) | *h^-^* *rad21-T262A-9PK-kan*^R^ *psm3*^K105NK106N^ *pph3*Δ::*hyg*^R^ | This study | 10473 | J.P.J. Lab. Strain collection |
| Strain, strain background (*S. pombe*) | *h^-^* *rad21-9PK-kan*^R^ *psm3*^K105NK106N^ *pph3*Δ::*hyg*^R^ | This study | 10505 | J.P.J. Lab. Strain collection |
| Strain, strain background (*S. pombe*) | *h^-^* *psl1-GFP-kan*^R^ | This study | 10513 | J.P.J. Lab. Strain collection |
| Strain, strain background (*S. pombe*) | *h^-^* *cdc10-129 leu1* *mis4-GFP-LEU2 pef1*Δ::*hyg*^R^ *rad21-T262E* | This study | 10644 | J.P.J. Lab. Strain collection |
| Strain, strain background (*S. pombe*) | *h^-^* *cdc10-129 leu1* *mis4-GFP-LEU2 rad21-T262E* | This study | 10645 | J.P.J. Lab. Strain collection |
| Strain, strain background (*S. pombe*) | *h^-^* *cdc10-129* *leu1-32 mis4-GFP-LEU2 pph3*Δ::*kan*^R^ | This study | 10658 | J.P.J. Lab. Strain collection |
| Strain, strain background (*S. pombe*) | *h^-^* *cdc10-129 rad21-9PK-kan*^R^ *pph3*Δ::*hyg*^R^ | This study | 10660 | J.P.J. Lab. Strain collection |
| Strain, strain background (*S. pombe*) | *h^-^* *cdc10-129 rad21-9PK-kan*^R^ *pph3*Δ::*hyg*^R^ *pef1*Δ::*ura4*^+^ *ura4-D18* | This study | 10666 | J.P.J. Lab. Strain collection |
| Strain, strain background (*S. pombe*) | *h^-^* *cdc10-129* *leu1-32 mis4-GFP-LEU2 pph3*Δ::*kan*^R^ *pef1*Δ::*hyg*^R^ | This study | 10668 | J.P.J. Lab. Strain collection |
| Strain, strain background (*S. pombe*) | *h^-^* *rad21-T262A-9PK-kan*^R^ *psm3*^K105NK106N^ *pph3*Δ::*hyg*^R^ *pef1*Δ::*nat*^R^ | This study | 10670 | J.P.J. Lab. Strain collection |
| Strain, strain background (*S. pombe*) | *h^+^* *pph3*Δ::*hyg*^R^ *psm3^K105NK106N^ mis4^P810A^* | This study | 10754 | J.P.J. Lab. Strain collection |
| Strain, strain background (*S. pombe*) | *h^+^* *pph3*Δ::*hyg*^R^ *psm3^K105NK106N^ mis4^T808P^* | This study | 10755 | J.P.J. Lab. Strain collection |
| Strain, strain background (*S. pombe*) | *h^+^* *pph3*Δ::*hyg*^R^ *psm3^K105NK106N^ mis4^P808I^* | This study | 10758 | J.P.J. Lab. Strain collection |
| Strain, strain background (*S. pombe*) | *h^+^* *pph3*Δ::*hyg*^R^ *psm3^K105NK106N^ mis4^Q822K^* | This study | 10759 | J.P.J. Lab. Strain collection |
| Antibody | Mouse monoclonal anti-V5 | BioRad | Cat# MCA1360 | (1:1000) |
| Antibody | Mouse monoclonal anti-HA 16B12 | Covence | MMS-101P | (1:2000) |
| Antibody | Mouse monoclonal 6X-HIS Tag (E3D10H2/E3) | ThermoFisher Scientific | MA1-135 | (1:5000) |
| Antibody | Mouse monoclonal anti-FLAG M2 | SIGMA | F1804 | (1:3000) |
| Antibody | Rabbit monoclonal anti-thiophosphate ester antibody | Abcam | ab133473 | (1:2000) |
| Antibody | Rabbit polyclonal anti-histone H3 | Abcam | ab1791 | (1:2000) |
| Antibody | Rabbit polyclonal anti-GFP | invitrogen | A11122 | (1:1000) |
| Antibody | Mouse monoclonal anti-GFP | Roche | Cat. No.11814460001 | (1:1000) |
| Antibody | Mouse monoclonal anti-tubulin antibody TAT1 | PMID:2606940 |  | (1:10000) |
| Antibody | Rabbit polyclonal anti-S.pombe Rad21 | PMID: 21300781 |  | (1:2000) |
| Antibody | Rabbit polyclonal anti-S.pombe Psm1 | PMID:  21189291 |  | (1:5000) |
| Antibody | Rabbit polyclonal anti-S.pombe Psm3 | PMID: 21300781 |  | (1:1000) |
| Antibody | Rabbit polyclonal anti-S.pombe Psm3-Ac | PMID: 21300781 |  | (1:1000) |
| Antibody | Rabbit polyclonal anti-S.pombe Rad21-T262P | This study |  | (1:1000) |
| Sequence-based reagent | TTGAAAAACACTGAAGCCATTG | This study | 438_fw | qPCR primer |
| Sequence-based reagent | ATTCATGCAGTCACGTCCAA | This study | 438_rev | qPCR primer |
| Sequence-based reagent | CGGGAAGTTACGAGCACATT | This study | tRNA-L_fw | qPCR primer |
| Sequence-based reagent | GGTTCCGATGGTCCTGATTA | This study | tRNA-L_rev | qPCR primer |
| Sequence-based reagent | TCGGGGTTTTTCACAATCAT | This study | imr2-L_fw | qPCR primer |
| Sequence-based reagent | CAGCACATCGACCAGGATTA | This study | imr2-L_rev | qPCR primer |
| Sequence-based reagent | AGGGTACGTTTAATTCTTGGTTTT | This study | cc2_fw | qPCR primer |
| Sequence-based reagent | ACGCATCACAAAGCAGTACA | This study | cc2_rev | qPCR primer |
| Sequence-based reagent | AAGGCCCACGTTGTTTGAAA | This study | dg2-R_fw | qPCR primer |
| Sequence-based reagent | TGGCATCAAATACTGCAAGGT | This study | dg2-R_rev | qPCR primer |
| Sequence-based reagent | GAGGACAGCATTGGCTGTTT | This study | tRNA-R_fw | qPCR primer |
| Sequence-based reagent | TGTCTGAAATCTCAACCATATTCAA | This study | tRNA-R_rev | qPCR primer |
| Sequence-based reagent | AGCAAAAGCACCGACTTCAT | This study | 1806_fw | qPCR primer |
| Sequence-based reagent | TTCAAAGCTGCTCTCCCATT | This study | 1806_rev | qPCR primer |
| Sequence-based reagent | GAAACCATTGTTTTAAGGCAACTT | This study | 2898_fw | qPCR primer |
| Sequence-based reagent | TCGGAACCTCTTCATTGCTT | This study | 2898_rev | qPCR primer |
| Sequence-based reagent | CAATTTCTTCTCTCGCTGTTGC | This study | 3323_fw | qPCR primer |
| Sequence-based reagent | TTGCGGCATCACACAATTGA | This study | 3323_rev | qPCR primer |
| Sequence-based reagent | ACGGACCAAGGAGTCTAGCA | This study | 28S_fw | qPCR primer |
| Sequence-based reagent | GTTCCCACCTGCATTCACTT | This study | 28S_rev | qPCR primer |
| Sequence-based reagent | TCTCTCATTTTCCATTGAACCA | This study | NTS_fw | qPCR primer |
| Sequence-based reagent | TTCAGGGTCGGTAGAGTCAGA | This study | NTS_rev | qPCR primer |
| Sequence-based reagent | ATGCTTTGGCCACTGTTCCT | This study | Tel1-R_fw | qPCR primer |
| Sequence-based reagent | TCAAAACCGCAAAAACGATG | This study | Tel1-R_rev | qPCR primer |
| Sequence-based reagent | ACCACAAATGGAGCCAAAAG | This study | ars3004_fw | qPCR primer |
| Sequence-based reagent | TTGCATCCAAGGCATCATAA | This study | ars3004_rev | qPCR primer |
| Commercial assay or kit | ChIP DNA Clean&Concentrator | Zymo research | D5205 |  |
| Commercial assay or kit | ChIP-Adembeads ProtA | Ademtech | 4242 |  |
| Commercial assay or kit | ChIP-Adembeads ProtG | Ademtech | 4342 |  |
| Commercial assay or kit | Absolute qPCR SYBR Green Mix | ThermoFisher Scientific | AB-1158/B |  |
| Commercial assay or kit | Reagents for coupled transcription/translation reactions | biotechrabbit | BR1402002 |  |
| Commercial assay or kit | ProtA µMacs | Miltenyibiotec | 130-071-001 |  |
| Commercial assay or kit | ProtG µMacs | Miltenyibiotec | 130-071-101 |  |
| Chemical compound, drug | ATP-γ-S | SIGMA | A1388 |  |
| Chemical compound, drug | p-Nitrobenzyl mesylate (PNBM) | Abcam | ab138910 |  |
| Chemical compound, drug | Anydrotetracycline hydrochloride | SIGMA | 37919 |  |
| Chemical compound, drug | 1-NA-PP1 | CAYMAN Chemicals | 10954 |  |
| Chemical compound, drug | Hydroxyurea | SIGMA | H8627 |  |
| software, algorithm | Prism | GraphPad |  |  |
| software, algorithm | Metamorph | Metamorph |  |  |
| software, algorithm | ImageJ | ImageJ |  |  |
| software, algorithm | FlowJo | FlowJo |  |  |
